# A descriptive qualitative case study of the experiences, perceptions and attitudes of pregnant women on Unguja island, Zanzibar, towards antischistosomal treatment

**DOI:** 10.1016/j.actatropica.2021.106143

**Published:** 2022-01

**Authors:** Eleanor De Rosa, Bobbie Person, Stefanie Knopp, Juma Muhsin, Jameelat Harriet Lyimo, Fatma Kabole, David Rollinson

**Affiliations:** a No affiliation; b Consultant of the Schistosomiasis Consortium for Operational Research and Evaluation, University of Georgia, Atlanta, Georgia, USA; c Swiss Tropical and Public Health Institute, Socinstrasse 57, 4051 Basel, Switzerland; d University of Basel, Petersplatz 1, 4003 Basel, Switzerland; e Neglected Diseases Program, Ministry of Health, P.O. Box 236, Zanzibar Town, Unguja, United Republic of Tanzania; f Wolfson Wellcome Biomedical Laboratories, Department of Life Sciences, Natural History Museum, Cromwell Road, London SW7 5BD, United Kingdom

**Keywords:** Control, Elimination, Education, Intervention, Mass drug administration, Praziquantel, Pregnancy, Qualitative research, Schistosomiasis, Zanzibar, HIV/AIDS, Human immunodeficiency Virus/Acquired immune deficiency syndrome, MDA, mass drug administration, NTD, neglected tropical diseases, SCORE, Schistosomiasis Consortium for Operational Research and Evaluation, TVZ, Television Zanzibar, USFDA, United States Food and Drug Administration, WHO, World Health Organisation, ZAMREC, Zanzibar Medical Research Ethics Committee, ZEST, Zanzibar Elimination of Schistosomiasis Transmission

## Abstract

Many countries exclude pregnant and lactating women from mass drug administration (MDA) programmes with praziquantel against schistosomiasis due to historic safety concerns over drug use during gestation and breast feeding. More than 10 years of empirical evidence from the field and a growing body of dedicated research has prompted the World Health Organisation and schistosomiasis control initiatives to advocate the inclusion of this vulnerable group into MDA. This qualitative descriptive case study explored, over a five-week period, the subjective experiences, perceptions, opinions, and attitudes of pregnant women attending government supported clinics on Unguja island, United Republic of Tanzania, towards praziquantel use during pregnancy in MDA programmes. The aim of the study was to identify and determine how to overcome potential barriers to effective use of MDA medications during pregnancy. Additionally, it was to determine trusted communication channels for future messaging and discover behavioural and community opportunities to increase participation of pregnant women in future MDA efforts. A 60 min, semi-structured qualitative interview was undertaken with 25 pregnant women recruited from 4 health centres on Unguja along with testing for *Schistosoma haematobium* infection. Using a modified-grounded theory approach, narrative data were transcribed, coded and analysed using a thematic analysis of the emergent themes. Women reported that they rely on traditional home remedies to stay healthy during pregnancy. Influenced by their mothers, husbands and neighbours, women predominately made medication choices during pregnancy and breastfeeding based on what they heard at home. Most women had been excluded from government MDA programmes in the past due to pregnancy. Women valued healthcare services for antenatal education and pregnancy advice. Women reported they would trust and follow direction from healthcare providers about taking praziquantel during pregnancy. Antenatal clinics offer an excellent opportunity to educate and expand praziquantel treatment to this cohort. Efforts should be augmented with training for providers and behavioural education for the community as a whole and family members of pregnant women.

## Highlights

• Women were enthusiastic about keeping healthy during pregnancy.
• Women strongly valued high quality antenatal care and were encouraged to attend clinics by their families and community members.
• Women demonstrated poor retention of knowledge about schistosomiasis from school.
• The majority of women interviewed had missed mass drug administration rounds of praziquantel due to pregnancy or concerns about side effects.
• Women were unanimously agreeable to taking praziquantel during pregnancy if advised to do so by a healthcare professional.

## Graphical abstract

Women were enthusiastic to attend local antenatal health education and were unanimously agreeable to taking praziquantel against urogenital schistosomiasis during pregnancy if advised to do so by a healthcare professional.

Picture credit: Bobbie Person

**Figure:**
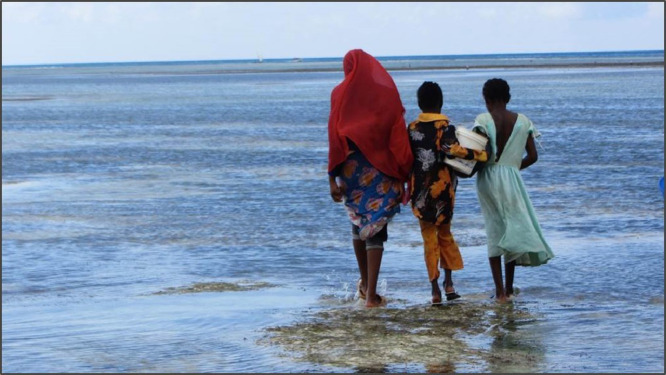

## 1 Introduction

Schistosomiasis is a water-borne parasitic disease caused by blood flukes of the genus *Schistosoma*. Infection causes a chronic and debilitating disease. In 2017, it was estimated that infection cost 1.43 million disability-adjusted life years (GBD 2017 DALYs and HALE Collaborators). *Schistosoma haematobium* causes the urogenital form of the disease and is endemic to Unguja island, Zanzibar, United Republic of Tanzania (Goatly and Jordan, 1965; McCullough and Krafft, 1976; Mgeni et al., 1990; Stothard et al., 2002a, 2000).

Sequelae of the disease include anaemia, sexual dysfunction, undernutrition and impairment of growth and cognitive development (Friedman et al., 2007; Godlove Bunda et al., 2019; Mpairwe et al., 2011; WHO, 2002). In women, involvement of the reproductive tract, placenta and umbilical cord has been demonstrated (Friedman et al., 2007; Sturt et al., 2020). Female genital schistosomiasis is likely the most neglected gynaecological condition in Sub-Saharan Africa and a cofactor for HIV/AIDS (Friedman et al., 2018; Gadoth et al., 2019; Godlove Bunda et al., 2019; Sturt et al., 2020).

The advent of praziquantel in the early 1980s created a safe and effective, single-dose anthelminthic for the treatment of schistosomiasis (Frohberg, 1984; Olds, 2005). Current policy advocates its use in mass drug administration (MDA) programmes. This approach, combined with behavioural change and intermediate snail host control, are likely to be the mainstays of schistosomiasis control for the foreseeable future (Fenwick et al., 2009; Friedman et al., 2007; Knopp et al., 2019a).

Praziquantel is listed by the United States Food and Drug Administration (USFDA) as “Pregnancy Category B”, which indicates that the drug is presumed safe based on animal studies, but has never been proven to be safe in adequate studies of pregnant humans (Friedman et al., 2007; Salawu and Odaibo, 2014). Despite a number of studies more recently suggesting praziquantel's safety, historical guidance has meant that pregnant women and, often women of childbearing age, have been excluded from national control programmes (Friedman et al., 2018; Ndibazza et al., 2010; Olveda et al., 2016). These women are therefore both extremely vulnerable to infection and a potential reservoir of parasites within the population.

The potential damage caused by infection in the pregnant mother is far-reaching, considering not only the effect on the foetus and impact on maternal health, but also considering her social role in terms of protecting other children from exposure and contributing to community dynamics and understanding of the disease (King and Dangerfield-Cha, 2008).

More than 10 years of clinical experience and evolving research from the field suggests a low potential toxicity of praziquantel in pregnancy (Frohberg, 1984; Olds, 2005; Olveda et al., 2016). The latest guidance from the World Health Organisation (WHO) advocates that all women be included in national coverage for schistosomiasis control (WHO, 2002).

Our qualitative descriptive case study explored, over a five-week period, the subjective experiences, perceptions, opinions, and attitudes of pregnant women attending government supported clinics on Unguja island, United Republic of Tanzania, towards praziquantel use during pregnancy in MDA programmes. It was conducted as part of the qualitative investigations for behavioural change carried out within the cluster randomized trial of the Zanzibar Elimination of Schistosomiasis Transmission (ZEST) project, supported by the Schistosomiasis Consortium for Operational Research and Evaluation (SCORE) (Knopp et al., 2012, 2019b; Person et al., 2016). The study presented here explored the thoughts of pregnant mothers surrounding praziquantel use during pregnancy, their knowledge of the disease and their attitudes towards participating in future rounds of MDA.

## 2 Methods

### 2.1 Ethical considerations

This study was conducted in July and August 2015 within the ZEST project. The ZEST study received ethical approval from the Zanzibar Medical Research Ethical Committee in Zanzibar, United Republic of Tanzania (ZAMREC, reference no. ZAMREC 0003/Sept/011) and is registered with the International Standard Randomized Controlled Trial Number register (ISRCTN48837681).

All participants of this qualitative case study provided written, informed consent for their participation. All participants were informed that their decision to take part in the study would not affect any future care they received. The study was overseen by a doctor from the Zanzibar Ministry of Health working within the Neglected Tropical Diseases (NTD) Programme in Zanzibar.

### 2.2 Study setting

The study took place on Unguja island, Zanzibar, the southern main island of the Zanzibar archipelago which forms part of the United Republic of Tanzania. The predominant language is Kiswahili and the main religion is Islam. In 2015, Unguja island was composed of 6 districts which are locally governed by leaders of smaller administrative units called shehias. The elected leader in each unit is referred to as the sheha who manages local administration (Pennance et al., 2016).

Urogenital schistosomiasis has long been considered a public health problem in Zanzibar (Knopp et al., 2012, 2019a, 2019b,; McCullough and Krafft, 1976; Mgeni et al., 1990; Stothard et al., 2002b, 2009). However, regular anthelminthic treatment with praziquantel in the 1990s and early 2000s and probably also a general improvement of the socio-economic status of many people have reduced infection levels with *S. haematobium* in the at-risk population (Guidi et al., 2010; Knopp et al., 2010; Montresor et al., 2002; Stothard et al., 2009). The ZEST project had the ultimate goal to eliminate urogenital schistosomiasis as a public health problem on the two islands of Zanzibar (Unguja and Pemba) and to interrupt transmission of the disease in the longer term (Knopp et al., 2012, 2019a, 2019b; Pennance et al., 2016). An associated cluster-randomized trial aimed to assess biannual MDA, applied alone or in combination with either snail control or behaviour change interventions for the reduction of *S. haematobium* infection prevalence and intensity in children on Zanzibar (Allan et al., 2020; Knopp et al., 2012, 2019a, 2019b; Person et al., 2016a, Person et al., 2016b).

### 2.3 Study design and data collection

Our study was designed as a descriptive qualitative case study, typically used when small numbers of cases are being explored (Crowe et al., 2011; Harrison et al., 2017). Qualitative research is typically conducted through semi-structured interviews, which are characterized by open-ended questions resulting in data of narrative text rather than numbers (Punch, 2013). In our study, in-depth interviews were used to better understand pregnant women's subjective experiences, perceptions, opinions, and attitudes towards the possibility of being administered medications in future MDA efforts given the historical precedent of being excluded in previous MDA campaigns.

**Fig. 1:**
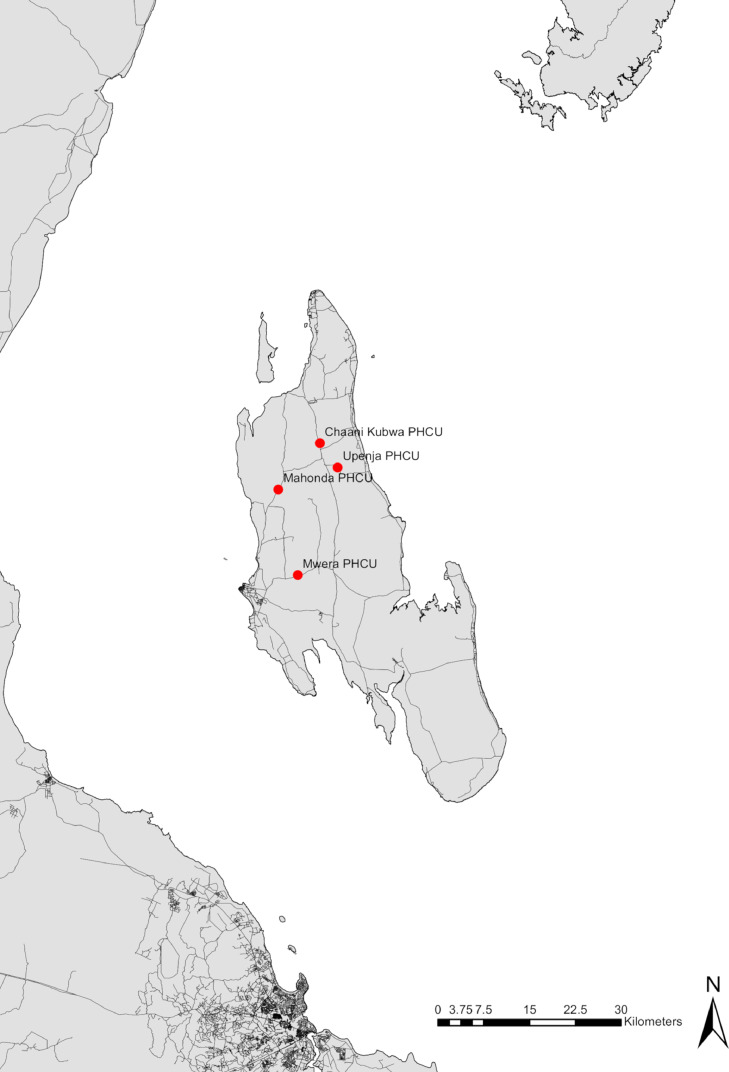
Location of the four study health facilities on Unguja island, United Republic of Tanzania.

At the beginning of the study, a semi-structured topic guide, along with consent leaflets was developed in collaboration with the senior social scientist and local ZEST behavioural team, translated into Kiswahili, pre-tested and revised post pre-testing to address clarity and linguistic nuances due to translation. Subsequently, over a 5-week period, pregnant women from 4 geographically discrete government health centres of Mwera, Mahonda, Upenja and Chaani (Fig. 1) were purposively selected on the day of interviews and invited to participate in the study. Each in-depth interview took 60 min in total, which included provision of study-related information, signing their consent form, being weighed, and measured, and invited to provide an own urine sample. The urine samples were examined for *S. haematobium* infection in the laboratory of the Zanzibar NTD Programme. Urine samples were shaken well and 10ml of each sample was filtered using a plastic syringe and filter holder containing a 13mm polycarbonate filter with a mesh size of 20.0 micron (Sterlitech, Kent, WA, USA). All *S. haematobium* eggs trapped on the filter of each participant were counted using a light microscope and recorded. Women who tested positive for *S. haematobium* infection were contacted and offered referral back through their health centres for counselling and treatment with praziquantel.

### 2.4 Data coding and analysis

A Conventional Qualitative Framework Method Thematic Analysis was used to identify and extract themes from the qualitative in-depth interview data (Gale et al., 2013; Hsieh and Shannon, 2005). Data were scrutinised to identify commonalities and contradictions, to draw explanations from clusters of themes. Answers were divided into cases and further subdivided into individual participant narrative quotes. The quotes were then analysed by the primary data coder (EDR) and reviewed by the senior social scientist (BP). Unrestricted open coding was used which was then used to assign categories to organise and manage the data. Categories were grouped into clusters around interrelated ideas and concepts, allowing data abstraction and formulation of final themes. With regard to the parasitological laboratory data, all women who had at least one *S. haematobium* egg identified in their urine sample were considered *S. haematobium*-positive.

## 3 Results

### 3.1 Study population

During the 5-week study period, a total of 25 women were interviewed. The in-depth interviews from the first 5 participants were used as a pilot to determine if any modifications were needed to the semi-structured interview topic guide. Since only minor modifications were needed the resulting data were included in the final data analysis.

Seventeen of the 25 participants were 20 to 30 years old and 20 had completed their primary and secondary school education. Half of the women were employed; half were housewives and half were carrying their third or more pregnancy. Five women had lost children in previous pregnancies or as infants. Most of the women were in their third trimester at the time of the study. As indicated in Table 1, one participant was recruited from Chaani, two from Upenja, eight from Mahonda and 14 from Mwera health centres.

**Table 1:** Characteristics of study participants on Unguja island, United Republic of Tanzania.

*Demographic*	Number of pregnant women (%)
*Age (years)*	
<20	5 (20%)
20–30	17 (68%)
31–40	3 (12%)
*Education level*	
Primary education	5 (20%)
Secondary education	20 (80%)
*Religious orientation*	
Christian	2 (8%)
Muslim	23 (92%)
*Co-wife* (polygamous)	1 (4%)
*Employment*	
Housewife	14 (56%)
Employed	11 (44%)
*Gravida*	
<3	12 (48%)
>3	13 (52%)
*Gestation at time of study*	
Week 1–12	1 (4%)
Week 13–26	5(20%)
Week 27 to term	19 (76%)
*Gestation at time of First clinic attendance*	
Week 1–12	6 (24%)
Week 13–26	17 (68%)
Week 27 to term	2 (8%)
*Health centre*	
Chaani	1 (4%)
Mahonda	8 (32%)
Mwera	14 (56%)
Upenja	2 (8%)
*Residency: rural versus urban*	
Rural	24 (96%)
Urban	1 (4%)
S. haematobium *infection*	
Positive	4 (16%)
Negative	21 (84%)

One participant described symptoms of pre-eclampsia and consented to referral through the healthcare system for treatment. She was included in the final analysis.

Four women had positive egg counts for *S. haematobium*. They all consented for referral and praziquantel treatment within the recruitment clinics. Three among the four women lived in Mwera district, and one woman was from Chaani.

### 3.2 Women's perceptions of health in pregnancy

Women unanimously described home solutions to keeping healthy during pregnancy through the use of a careful diet. One woman said, “*We are told to eat everything to promote the health of the mother and the child.*” (P06). Women perceived that food such as fresh fruit, vegetables and protein-rich meals could be therapeutic in managing concerns they had about health in pregnancy, *“I take the good, high-protein foods to increase the blood protein. I take food with a lot of water in [...] to clean the urine.*” (P22). Another woman reported, “*I take all food but the salt [...] I worry about pressure* (high blood pressure).” (P24). Women felt exercise would be helpful, “*If inactive, delivery is delayed so it is good to exercise.*” (P06).

Women's perceptions of how to have a healthy pregnancy were influenced by their neighbours, families, and local clinic professionals. *“I learn about food from my neighbours. My sister also teaches.”* (P22)*, “[we do not learn how to be healthy during pregnancy] in the community, [we learn] in the clinic. My sisters and neighbours talk a little in the evening.”* (P23), *“The sheha meeting.”* (P21), *“My mother teaches me to take the food and other drugs, like mfagio (herbal tea). My husband also teaches me; he learns from the hospital.”* (P16).

Health concerns in pregnancy predominately surrounded anaemia and problems with high blood pressure. They were based upon personal experience and what women were taught in health centre antenatal sessions *“[it is important...] to keep the HB* (haemoglobin) *up because it helps for delivery [...]. I learnt this from the doctor in hospital- we were all educated together by the doctors here.”* (P16). Another woman told us, “*High HB keeps you and the baby healthy and allows you to keep up activities.*” (P11). While, women who had lost children in the past advocated attending clinic early to detect potential health problems, many women reported a delay in recognizing that they were pregnant until they sought a test from hospital or advice from a family member following interruption of menstruation.

### 3.4 Medicine in pregnancy

Women reported that they took medication because they were either unwell or had been told to by a healthcare worker. There was little awareness or concern surrounding potential harmful effects of medication during pregnancy or whilst breastfeeding, “*If I am sick, I will take medicine.”* (P10). *“My husband told me not to take the medicines as they are not good for pregnant women. If very, very sick, then it is better to go to hospital and take advice from doctors, not neighbours or family.”* (P08).

All but one of the women who had previously lost a child, commented that medication in pregnancy was not good unless advised by a healthcare professional, *“No. It's not good to eat drugs, if you're not sick. If you are sick, it's better to go to hospital for doctor to request and go to pharmacy to get. Not to go to pharmacy alone without doctor's advice.”* (P20).

Some women felt that medication at any time, as given by a health professional, could only confer a positive effect on health, *“Yes. You prevent pain and sometimes infection.”* (P09). Women reported being less troubled about taking medication whilst breastfeeding without advice from a healthcare worker, *“For good health for the baby and the mother and after delivery it won't affect the baby.”* (P11).

### 3.5 Maternal health service provision

Most women valued the ability to access clinic services, “*I came here as all the services are free and there are many checks done here. The staff are very good also and the service is good.”* (P24). Women reported value in having a thorough, scheduled examination with the use of instruments to examine them such as auscultation of the baby's heart rate and blood pressure monitoring. They felt the service educated and prepared them for birth, and helped them avoid complications, *“the service is good. I like the scales for the baby, pregnant mother and all the activities for the clinic.”* (P25). Many women planned to give birth in these local clinics whilst first time mothers were referred to regional centres.

### 3.6 Knowledge of schistosomiasis

All but one participant (P13) recognized the term schistosomiasis, or ‘kichocho’ (the Kiswahili word for schistosomiasis), and most identified that the pathogen or ‘Vidudu’ was in the river. Some women talked about ‘dirty water’ and a propensity for male children to become infected, *“Kichocho is a disease in the river or dirty water. In the river is Vidudu. Vidudu is transmission of kichocho. Special for the boys if any activity of fishing, swimming, washing clothes.”* (Ma20). Many women talked about the snail, or ‘konkono’, *“Kichocho is a disease that lives in river and water. In the river is konokono. The konokono transmits kichocho. If we go in the river, we get kichocho. I do not know the signs and symptoms of kichocho.”* (P19). The most predominant symptom of the disease reported in interviews was blood in the urine and abdominal pain, *“First urine blood and then lower abdominal pain. Then medicine for kichocho we get at hospital only. In the river are konokono for the transmission of kichocho.”* (Up17). Those women who tested positive for *S. haematobium* eggs were equally aware.

### 3.7 Learning about kichocho

The majority of women had learned about schistosomiasis at school although some commented that this was a long time ago, “*School – I can't remember what they said, it was 2005.*” (P22), *“School when I was in standard 5 or 6. I still remember.”* (P04). Women also learnt about kichocho through the media; TV Zanzibar (TVZ) and radio, “*I learned about it on TV and radio.*” (P23), “*I learned from neighbours, sometimes on the radio. It is an endemic disease. Radio-special if MDA time – any day, any time.*” (P15). A few women recalled that they had heard about schistosomiasis in the shehia or from neighbours, “*School, and I learnt through the community meeting [...] several times.*” (P16). Women were unanimously keen to learn more about kichocho and felt the hospital or community would be the place to do so, “*Yes. It is very important to learn more about kichocho in the wider perspective. All about kichocho first and then the children will still go to the river. So, bring the information to the community and teach all to prevent it.*” (P18). They felt schistosomiasis was an issue for all the family as “*Water is for all.*”, “*I would be interested in learning more about the medicine. Some neighbours say the medicine is not good and it's better to have good information about kichocho medicine.*” (P07). All but one participant expressed that kichocho was an important issue for the whole family, *“Yes it's a big problem. Because this kichocho is a big problem in the community as there is medicine for kichocho but no way to cut off the kichocho. We have had it for many years in Zanzibar, but the ways to stop kichocho have not stopped kichocho.”* (P12). All but the same one participant wanted to learn more about kichocho, “*Yes, all about kichocho and treatment and prevention.*” (P02). The participant who did not answer positively previously stated that she had never heard of ‘kichocho’ before.

### 3.8 Experience with praziquantel

A total of 10 women stated that they had heard that the medication should not be taken in pregnancy. They had learned this from multiple sources, most commonly from MDA drug distributors, mothers and neighbours, *“the drug distributor said, ‘this drug is not good for pregnant mothers.”* (P12)*.* “*My mother said, do not take drugs as it complicates (the pregnancy), but with the doctor's advice, mother said it was good.”* (P03), *“My neighbours say, “not to eat kichocho medicine as it is contraindicated for pregnant mother.”* (P13). Two participants explained they had decided themselves not to take the medication, *“You shouldn't take the drugs, but no-one told me this. I taught myself.”* (P25), “*I think kichocho medicine may affect the baby.*” (P21). Both of these women were in their 6^th^ pregnancy, and both said they did not avoid anything but medication during pregnancy. Neither had lost a child. Of the 5 participants who tested positive for schistosomiasis, none of them had been treated recently, due to advice from drug distributors or their mother. Two of the 5 women who had previously stated they would not take medication at all during pregnancy said they had not heard pregnant women should not take kichocho medicine. Of the remaining three, one was influenced by her mother not to take the drug, one by her neighbour and one by a drug distributor.

Two women had heard rumours about praziquantel being harmful, “*The medicine is not good as it reduces the number of babies. I heard this from neighbours.”* (P12). *“Some of the neighbours say it is very powerful and will reduce child growth, but other neighbours say it is a good drug and it will treat other diseases.”* (P06). They had both taken the medicine some years ago when not pregnant.

### 3.9 Taking the tablets and trusted sources of information

When women were told that the WHO had come together and decided that praziquantel was a safe and helpful medicine to take in pregnancy, all but one said they would take the tablet to ensure good health for their babies, “*Yes, to prevent the mother and baby being sick.*” (P20), “*Yes, because of good information and advice, then there is no problem.*” (P19). The participant who would not take it said *“No, as it may cause abortion and I will feel worried.”* When prompted, *“If a doctor told you it was safe, would you take it?” “Yes.”* She was a first-time mother who had recently moved from another district, taken praziquantel at school and learnt from her mother about pregnancy. She had not attended clinic before (P07). Ten of the women interviewed wanted the advice of a ‘doctor’ to reassure them first. Many of the other women reported that they would accept advice from any health professional from the clinic, “*If the doctor advised first.*” (P01), “*If the doctor advised.*” (P04). Some reported that they would like to test positive before taking the medication, “*Yes. First we check and then give me the medicine.*” (P12), *“No problem if you're sick.*” (P03).

When asked what the best way was to persuade families that praziquantel was a good medication to take during pregnancy, women explained that community education would be most helpful. Many felt that information given from a healthcare worker in the community would be best, *“It's best to go to the community meeting and then to educate all about kichocho, and not one-by-one. The sheha will announce the meeting before.”* (P18), “*Call them or hold a meeting. […] it's better to go to the sheha, or community.*” (P14), *“Take everyone, including children together and talk about kichocho. It depends, sometimes in the house and sometimes in the village meeting.”* (P09). Some suggested that leaflets could be helpful, *“Give a photocopy of all education about kichocho and give it to the community. If anyone cannot read, the partner that does should take responsibility to learn and educate the other.”* (P24). The hospital was also a choice place for women to learn, *“Mother education and hospital group education sessions. Some people like the radio.”* (P03*), “I do not know, they should come to the hospital like Mwera.”* (P04).

## 4 Discussion

Despite regular MDA and additional elimination interventions, the risk of acquiring a schistosome (re-)infection remains high in certain areas in Zanzibar (Trippler et al., 2021). This study assessed the subjective experiences, including knowledge, perceptions, opinions, and attitudes of pregnant women towards taking praziquantel during their pregnancy. The purpose of the study was to assess what would be necessary to incorporate pregnant women into future MDA programmes, with the aims of improving disease control and of sustaining and accelerating elimination efforts.

Women were enthusiastic to maintain optimal health in pregnancy and valued high quality antenatal care. Women were encouraged to attend clinics by their families and community members. Outside of such clinics, their knowledge was mainly accrued through word of mouth within communities, from family and neighbours and through health educational lessons at local health centres. Although women frequently referenced school as the location where they first learned about schistosomiasis and health in pregnancy, women commented on not retaining information over time and their children not relaying learning to the home. This suggests that health education could be more effective if supported by the sheha and community behavioural education sessions for all to attend. Given the number of years that MDA has been administered in these communities and the consistent exclusion of pregnant women in these efforts women suggested that health education and services could be further augmented by written leaflets and announcements on the radio/TV. The availability of health education services is likely to have a positive effect in decreasing case fatality due to pregnancy-related complications and improving community health (Acharya and Cleland, 2000; Seward et al., 2017). These services offer the MDA programme a potential target to augment their efforts to ensure that extending MDA coverage to pregnant women is successfully implemented.

Women managed concerns about pregnancy through the use of home remedies and careful diet which reflects practices in other parts of Tanzania and Uganda (Mubyazi et al., 2005; Parker et al., 2008). There was limited understanding by women that medications could be detrimental during pregnancy or breastfeeding. Despite this, most women felt that medication during pregnancy was only acceptable if there was a disease to be treated and the medication was offered by a health professional. Research suggests that pregnant women, especially from a less educated background, tend to overestimate the risk of medication in pregnancy which may act as a barrier to MDA implementation in this group (Nordeng et al., 2009; Zaki and Albarraq, 2014). Women's preferred communication channel for receiving information about praziquantel for use during pregnancy was from a doctor or other health professional at the clinic. They unanimously said that they could be reassured by trusted healthcare workers to take medication during pregnancy. Women were generally familiar with praziquantel but the majority had missed rounds due to pregnancy and had not taken praziquantel for some time. Drug distributors who are perceived by communities to be knowledgeable, influential and well-integrated are typically identified as being invaluable to the success of MDA programmes (Babu and Kar, 2004; Chami et al., 2017; Odhiambo et al., 2016; Parker et al., 2008). In our study, women often found that drug distributors lacked the knowledge to reassure them of safety in taking praziquantel during pregnancy. Some women chose not to swallow the medication despite accepting it from the drug distributor due to concerns about side effects, which is a consistent theme throughout MDA non-compliance research. Participant decisions surrounding taking praziquantel were largely led by community perception and their own experience with the drug, such as its side effects and the thoughts of family and neighbours, whose opinions play a significant role in the mother's decision making.

Interestingly, despite the fact that women may have previously been told that the medication was unsafe, there did not seem to be a tangible fear surrounding praziquantel when compared to other concerns within pregnancy such as anaemia and vaginal bleeding. Indeed, the concerns women had, appeared more to reflect the curriculum from health clinic antenatal classes and problems experienced by acquaintances. This supports evidence to suggest that fear around side effects is often outweighed by personal experience and the experiences of friends and family (Odhiambo et al., 2016). For some women it was important that they tested positive for schistosomiasis before taking praziquantel. This may be difficult to implement at MDA but would be possible during clinic visits. We demonstrated a large emphasis on community meeting and discussion with neighbours. Evidence highlights the important role of the community, identifying that integrated members are more likely to understand the disease, the role of MDA and are more likely to receive drugs by distributors (Chami et al., 2017). Some women in our study acknowledged that they do not attend shehia meetings. Our data suggest that these women may be drawn into community hubs and women's groups should they be perceived to offer quality health education.

Extending healthcare into communities has been demonstrated to foster trust and public health education, which offers opportunities to improve antenatal care and anthelminthic drug uptake by reinforcing and supporting MDA programmes. Trials identifying ways to improve medication uptake in other MDA programmes similarly demonstrated that high quality, community delivered initiatives, using well-trained and respected community health workers, significantly improved medication uptake (Babu and Kar, 2004; Inobaya et al., 2018; Krentel et al., 2013; Odhiambo et al., 2016; Parker et al., 2008; Talbot et al., 2008; Tuhebwe et al., 2015).

A potential barrier to successful implementation of MDA may have arisen from rumours surrounding praziquantel negatively affecting fertility and impairing foetal growth that stemmed from communities. Reassuringly, these comments were one-off rumours, and neither were expressed by more than one participant. Our case study was restricted to clinic sites, and it remains a concern that these rumours could be more prevalent in harder-to-reach populations not addressed in this study, such as mothers who do not attend antenatal clinics and may rely more on home remedies, community support and traditional home-birthing techniques. These populations are likely to have multiple risk factors for non-compliance such as lower socioeconomic status and relative social isolation (Chami et al., 2016). This population is a vital subgroup to interview for the benefit of all the schistosomiasis control and elimination activities on Unguja island, Zanzibar.

The qualitative nature of this descriptive study is not generalizable to the whole of Zanzibar. But the findings do suggest that including pregnant women in MDA efforts in these communities could be more successful than initially predicted if personally recommended by a trusted healthcare provider and supported by local health centres where schistosomiasis can be formally incorporated into the antenatal teaching syllabus and wider community health initiatives. Community health education sessions offer an opportunity to dispel rumours and support treating pregnant mothers through high-quality health education from healthcare workers they trust. This could work synergistically with existing drug distribution schemes to encourage community uptake and reinforce other schistosomiasis control techniques. It may also help to attract and integrate harder-to-reach populations with a higher burden of infection, facilitating far-reaching health benefits.

## 5 Conclusion

The women from Zanzibar interviewed in our study valued high quality health education and were keen to attend more pre-natal counselling from healthcare professionals to ensure their pregnancy runs smoothly. They valued the opinion of health professionals and high quality care and, as such, women seemed to be moving towards health-service based education with a growing emphasis on TV and radio to advertise and reinforce healthcare messages. They recognised urogenital schistosomiasis as a problem in the area and were keen to understand more to keep their families safe. They would be prepared to take praziquantel antenatally if advised by a healthcare professional.

## Funding information

This study received financial support from the University of Georgia Research Foundation Inc., which is funded by the Bill & Melinda Gates Foundation (https://www.gatesfoundation.org/) for these Schistosomiasis Consortium for Operational Research and Evaluation (SCORE; https://score.uga.edu/) projects (prime award no. OPP50816, sub-award no. RR374-053/4893206). SK received financial support by sub-award no. RR374-053/4893196. The funders of the study had no role in the study design, data collection and analysis, decision to publish, or preparation of the manuscript.

## CRediT authorship contribution statement

**Eleanor De Rosa:** Conceptualization, Methodology, Formal analysis, Investigation, Data curation, Writing – original draft, Writing – review & editing, Visualization. **Bobbie Person:** Conceptualization, Methodology, Formal analysis, Investigation, Data curation, Writing – review & editing, Supervision, Project administration. **Stefanie Knopp:** Conceptualization, Resources, Writing – review & editing, Supervision, Project administration, Funding acquisition. **Juma Muhsin:** Conceptualization, Writing – review & editing, Supervision. **Jameelat Harriet Lyimo:** Investigation, Writing – review & editing. **Fatma Kabole:** Resources, Writing – review & editing, Project administration. **David Rollinson:** Conceptualization, Resources, Writing – review & editing, Supervision, Project administration, Funding acquisition.

## Declaration of Competing Interest

Authors have declared that no competing interests exist.
